# Beyond Calories: Individual Metabolic and Hormonal Adaptations Driving Variability in Weight Management—A State-of-the-Art Narrative Review

**DOI:** 10.3390/ijms252413438

**Published:** 2024-12-15

**Authors:** Nikolaos Theodorakis, Magdalini Kreouzi, Andreas Pappas, Maria Nikolaou

**Affiliations:** 1 NT-CardioMetabolics, Clinic for Metabolism and Athletic Performance, 47 Tirteou Str., 17564 Palaio Faliro, Greece; nikolaostheodorakis1997@yahoo.com; 2 Department of Cardiology & Preventive Cardiology Outpatient Clinic, Amalia Fleming General Hospital, 14, 25th Martiou Str., 15127 Melissia, Greece; 3 School of Medicine, National and Kapodistrian University of Athens, 75 Mikras Asias, 11527 Athens, Greece; 4 Department of Internal Medicine, Amalia Fleming General Hospital, 14, 25th Martiou Str., 15127 Melissia, Greece; kreouzi.m@live.unic.ac.cy; 5 Department of Informatics and Telecommunications, National and Kapodistrian University of Athens, Panepistimioupolis, Ilisia, 15784 Athens, Greece; andreas@pappas.xyz

**Keywords:** obesity, weight management, weight loss, metabolism, precision nutrition, muscle mass, artificial intelligence, dominant energy balance model, calories in, calories out model, carbohydrate–insulin model

## Abstract

The global rise in obesity underscores the need for effective weight management strategies that address individual metabolic and hormonal variability, moving beyond the simplistic “calories in, calories out” model. Body types—ectomorph, mesomorph, and endomorph—provide a framework for understanding the differences in fat storage, muscle development, and energy expenditure, as each type responds uniquely to caloric intake and exercise. Variability in weight outcomes is influenced by factors such as genetic polymorphisms and epigenetic changes in hormonal signaling pathways and metabolic processes, as well as lifestyle factors, including nutrition, exercise, sleep, and stress. These factors impact the magnitude of lipogenesis and myofibrillar protein synthesis during overfeeding, as well as the extent of lipolysis and muscle proteolysis during caloric restriction, through complex mechanisms that involve changes in the resting metabolic rate, metabolic pathways, and hormonal profiles. Precision approaches, such as nutrigenomics, indirect calorimetry, and artificial-intelligence-based strategies, can potentially leverage these insights to create individualized weight management strategies aligned with each person’s unique metabolic profile. By addressing these personalized factors, precision nutrition offers a promising pathway to sustainable and effective weight management outcomes. The main objective of this review is to examine the metabolic and hormonal adaptations driving variability in weight management outcomes and explore how precision nutrition can address these challenges through individualized strategies.

## 1. Introduction

Achieving sustained weight loss is a formidable health goal, requiring not just the reduction in body fat but also the preservation of lean mass-a critical factor for metabolic health and functional capacity. Obesity rates are rising globally, with the age-standardized prevalence increasing from 4.6% in 1980 to 14.0% in 2019 [1]. Traditional weight management often hinges on the “calories in, calories out” model, a simple formula that suggests weight loss is as straightforward as burning more calories than consumed. While appealing due to its simplicity, this model has limitations, as initial success in weight loss often plateaus or reverses due to the body’s adaptive responses. The “calories in, calories out” model, also known as the dominant energy balance model, assumes that caloric expenditure and intake are accurately measurable and linearly related to weight loss [2]. However, calculating the caloric needs has notable limitations:

▪ Firstly, food energy labels are allowed a margin of variability, with calorie counts fluctuating by up to 20% according to regulatory standards; however, in practice, discrepancies can be as high as 60% [3,4].
▪ Secondly, not all calories affect the body equally. The carbohydrate–insulin model, according to a recently published perspective, proposes a different causative pathway for weight gain. Unlike the “calories in, calories out” model, the carbohydrate–insulin model suggests that it is not just calorie quantity but also the type of calories that plays a crucial role. For instance, 500 calories from whole-grain rice and 500 calories from white crystalline sugar impact the metabolism differently. Whole-grain rice, with a lower glycemic index (GI), is digested more slowly, causing a gradual rise in blood glucose and promoting satiety due to its high fiber content [4]. Additionally, low-GI foods like whole grains may lead to lower total calorie absorption, as some of their fiber and resistant starches pass through the digestive tract partially undigested. In contrast, a lower fiber, high-GI food like white crystalline sugar causes a rapid spike in blood glucose, leading to a quick insulin response that promotes lipogenesis and often stimulates hunger soon after [2]. This illustrates how food quality, not just quantity, influences weight management.
▪ The third and perhaps most significant limitation of the “calories in, calories out” model is the body’s adaptive responses to changes in energy intake, which are conceptually linked to the weight “set point” theory. According to this theory, body weight is maintained within a relatively stable range by a biological feedback system that adjusts food intake and energy expenditure [5]. However, this set point is not absolute and likely allows for a range of variability, influenced by factors such as metabolic adaptations, hormonal fluctuations, and environmental factors, such as exercise, nutrition, sleep, and stress. These adaptive processes include variations in the basal metabolic rate (BMR), differential energy storage mechanisms, and dynamic shifts in hormonal regulation, all of which alter energy balance in ways that a simple caloric model cannot fully capture. Factors such as body composition, genetic predispositions, insulin sensitivity, and individual hormonal profiles collectively drive these responses, creating unique weight outcomes for each person [6,7].

A head-to-head comparison of the key features of the “calories in, calories out” and carbohydrate–insulin models is presented in Table 1.

The above comparison underscores the insufficiency of a one-size-fits-all approach to weight management and highlights the need for personalized strategies tailored to an individual’s biological characteristics, especially in preserving lean mass and achieving sustainable weight management. Recent advancements in artificial intelligence (AI) and machine learning (ML) offer transformative potential for addressing this variability. By analyzing vast datasets—including genetic, hormonal, and lifestyle factors—AI can identify patterns and predict metabolic responses to various dietary and lifestyle modifications. These insights not only move beyond the traditional “calories in, calories out” paradigm but also pave the way for truly personalized and sustainable weight management strategies.

The aim of this review is to provide a comprehensive analysis of the metabolic and hormonal mechanisms that drive variability in weight management outcomes. By synthesizing existing evidence, this work introduces a novel perspective on individualizing weight management strategies using precision nutrition approaches. Specifically, the review highlights potential applications of advanced tools, such as nutrigenomics, AI, and indirect calorimetry, to tailor interventions to each individual’s metabolic profile. By moving beyond the traditional “calories in, calories out” model, this review emphasizes integrating metabolic science with emerging technologies, offering a unique and novel framework for addressing the limitations of conventional weight management paradigms. This approach lays the foundation for personalized, sustainable, and effective interventions.

## 2. Methods

This manuscript is a state-of-the-art narrative literature review synthesizing current knowledge on the metabolic and hormonal mechanisms underlying individual variability in weight management, as well as precision approaches for sustainable outcomes. A comprehensive literature search was conducted in PubMed and Scopus for studies published up to 1 November 2024. We utilized a wide range of keywords and Medical Subject Headings (MeSH) terms to ensure a thorough search, including but not limited to weight management, weight loss, weight gain, metabolism, hormonal variability, precision nutrition, obesity, caloric restriction, overfeeding, somatotypes, and adaptive thermogenesis. Boolean operators were applied to refine and optimize the search strategy. The inclusion criteria were (1) reviews or original research articles published in English up to 1 November 2024; (2) articles focusing on metabolic and hormonal mechanisms driving individual variability in weight outcomes; and (3) publications addressing precision nutrition and personalized approaches. Articles were initially screened based on their titles and abstracts to assess relevance. Subsequently, full-text reviews were conducted for studies meeting the inclusion criteria. To ensure comprehensive coverage, additional relevant articles were identified through a manual review of reference lists from selected studies. The findings were then synthesized to present an integrated and up-to-date perspective on the topic.

## 3. Classification of Somatotypes

The classification of somatotypes—ectomorph, mesomorph, and endomorph—provides an essential framework for understanding individual variability in weight management. While initially based on external physique, these body types correspond to distinct metabolic and physiological profiles that influence each person’s response to diet, exercise, and caloric changes. This approach highlights the diverse ways individuals manage fat storage, muscle development, and energy expenditure [8,9].

Ectomorphs, who are typically lean with a lower propensity for fat storage, often experience a naturally higher metabolic rate and reduced efficiency in energy storage. These individuals tend toward catabolism, which means they may struggle to gain or maintain lean mass, particularly during caloric restriction. Their lower baseline insulin levels lead to less efficient nutrient storage but promote higher energy expenditure, which aligns with their natural predisposition to a fast metabolism. Precision weight management for ectomorphs may include high-protein diets and resistance-based exercises to help preserve lean muscle during weight loss [8,9].

Mesomorphs, characterized by a muscular build, benefit from a balanced metabolic efficiency. This body type generally has a higher muscle mass, which supports an elevated BMR, aiding in the maintenance of lean mass during caloric deficit. Mesomorphs are typically responsive to diet and exercise changes, often achieving consistent and predictable weight loss outcomes. Their relatively balanced hormonal environment facilitates a stable energy balance, making them more adaptable to shifts in caloric intake and expenditure. Optimal weight management for mesomorphs may involve a balanced macronutrient intake and consistent strength training to maximize muscle preservation while supporting fat loss [8,9].

Endomorphs, with a tendency toward fat storage and efficient energy conservation, often face challenges in weight loss due to a lower baseline metabolic rate and a metabolic efficiency that favors energy storage. This predisposition, which might have been beneficial in times of food scarcity, can make fat loss more difficult, as endomorphs tend to store calories more readily even under moderate caloric intake. Their hormonal profile often features higher baseline insulin levels and a greater anabolic response, which favors fat storage. Additionally, higher leptin and cortisol levels may further encourage energy storage and hinder weight loss efforts. Effective strategies for endomorphs may involve a focus on low-GI diets, along with stress management techniques to modulate insulin and cortisol levels, thereby supporting a more favorable response to caloric restriction [8,9].

## 4. BMR and Resting Metabolic Rate

The BMR and resting metabolic rate (RMR) are critical metrics in energy expenditure, forming the foundation for understanding individual variations in weight maintenance, loss, and gain. BMR represents the energy required to sustain the body’s essential physiological functions at rest, including cellular processes, thermoregulation, and organ function. This rate, typically measured under strict conditions, provides insight into the minimum caloric needs necessary to maintain basic life processes. RMR is a closely related measure, representing the energy expenditure at rest in a non-fasting state. While RMR is generally easier to measure—via indirect calorimetry—and slightly higher than BMR, both rates serve as foundational indicators of metabolic efficiency and energy needs [10].

Body composition, specifically the ratio of muscle to fat mass, exerts a considerable impact on the BMR and RMR. Muscle tissue is metabolically active and demands more energy than adipose tissue, leading individuals with higher muscle mass to experience an elevated BMR and RMR. Consequently, individuals with a greater lean body mass typically have a higher baseline energy expenditure, which can be advantageous for weight management, as it provides a greater caloric buffer. In contrast, individuals with a higher proportion of fat mass often exhibit a lower BMR, which may predispose them to weight gain, especially under a surplus of calories [11].

## 5. Overview of Hormonal Control of Metabolism

The body’s handling of carbohydrates, fats, and proteins is orchestrated through complex metabolic and hormonal networks, which determine how each macronutrient is metabolized, stored, or converted depending on availability, caloric state, and individual metabolic profiles. Hormonal signals integrate with these metabolic pathways to promote or inhibit energy storage and expenditure across different tissues, creating a finely tuned balance in response to dietary intake [12,13,14,15,16,17,18,19,20,21,22,23,24]. The key effects of major hormones affecting the metabolism of carbohydrates, fats, and proteins are presented in Table 2.

Weight cycling, commonly referred to as “yo-yo dieting”, involves repeated cycles of weight loss followed by weight regain. There is a widespread belief that this pattern leads to adverse effects, such as increased fat accumulation, reduced resting metabolic rate (RMR), and diminished success in subsequent weight loss efforts. However, a systematic review of 23 studies examining the physiological effects of weight cycling paints a more nuanced picture. Most studies found no significant associations between weight cycling and changes in body weight, body mass index (BMI), fat mass (FM), or lean body mass (LBM). Additionally, no substantial evidence supported a negative impact on RMR. These findings suggest that for healthy individuals managing overweight or obesity, repeated weight loss attempts do not necessarily compromise metabolic health or body composition. This evidence underscores the importance of encouraging sustained weight management efforts without fear of detrimental effects from previous cycles [25].

## 6. Adaptations to Caloric Restriction

Caloric restriction invokes a suite of adaptive mechanisms involving adaptive thermogenesis, changes in appetite, alterations in hormonal and metabolic profiles, and changes in body composition. These adaptations represent a survival mechanism that enables the body to prolong energy reserves during food scarcity, albeit with notable inter-individual variability.

### 6.1. Adaptive Thermogenesis

Caloric restriction typically induces a decrease in the BMR and RMR, as the body initiates adaptive responses to conserve energy. This metabolic deceleration is often more pronounced in individuals who experience greater adaptive thermogenesis, a phenomenon driven by both hormonal shifts and autonomic nervous system adjustments. Adaptive thermogenesis occurs as a survival mechanism, helping the body endure periods of food scarcity by reducing energy expenditure. However, the magnitude of this adaptation varies widely across individuals, with some experiencing only minor changes in BMR, while others see significant reductions, complicating efforts to maintain a steady weight loss trajectory [26,27]. A milestone study by Leibel, Rosenbaum, and Hirsch (1995) examined how changes in body weight influence energy expenditure in obese and non-obese individuals. The findings revealed that a 10% weight reduction led to a significant decrease in total energy expenditure (6 ± 3 kcal/kg fat-free mass/day in non-obese subjects and 8 ± 5 kcal/kg/day in obese subjects, both *p* < 0.001) [28]. Notably, this reduction in RMR might persist even after regaining the lost weight, as highlighted by a six-year follow-up of the participants of The Biggest Loser study. This study showed that participants initially lost 58.3 ± 24.9 kg, reducing their RMR by 610 ± 483 kcal/day (*p* = 0.0004). Six years later, although most weight was regained, the RMR remained 704 ± 427 kcal/day below the baseline (*p* < 0.0001). Changes in body composition, including lower lean mass, as well as other complex factors might contribute to this phenomenon [29].

Caloric restriction reduces the circulating levels of triiodothyronine (T3), the active form of thyroid hormone, while thyroxine (T4) also drops. A reduction in T3 results from downregulation of the type II iodothyronine deiodinase enzyme, which normally converts T4 into T3 in peripheral tissues. Decreased T3 downregulates metabolic pathways across tissues, including liver, muscle, and adipose, by inhibiting the transcription of genes that stimulate ATP-consuming processes. Lowered T3 also reduces mitochondrial biogenesis, oxidative phosphorylation, and basal thermogenesis, impacting the energy expenditure of key tissues. As T3 levels decline, reverse T3 (rT3) levels increase. rT3 is an inactive form of T3 that binds to thyroid hormone receptors without activating them, thereby blocking T3’s effects. Elevated rT3 during caloric restriction reinforces a low metabolic state, lowering cellular ATP turnover and limiting energy output [26,27,30].

Furthermore, the sympathetic nervous system (SNS) modulates thermogenic responses via catecholamines like norepinephrine, which activate β-adrenergic receptors in brown adipose tissue (BAT). Under caloric restriction, SNS activity decreases, reducing norepinephrine release and β-adrenergic receptor stimulation, especially in BAT, where mitochondrial uncoupling protein (UCP1) activity drops. UCP1 typically dissipates energy as heat through uncoupled oxidative phosphorylation in BAT; therefore, its downregulation under caloric restriction further decreases energy expenditure [26,27,31].

Additionally, during caloric restriction, leptin levels decline proportionally to fat loss. This reduction signals an energy deficit to the hypothalamus, leading to decreased sympathetic output, lowering thermogenesis, and reducing BMR to conserve energy. Low leptin levels also reduce hypothalamic–pituitary–thyroid axis activity, further downregulating T3 levels and exacerbating metabolic slowdown [26,27].

### 6.2. Appetite Stimulation

As caloric restriction persists, hormonal signals drive appetite regulation, intensifying the body’s drive to seek food and restore energy reserves. Ghrelin, primarily produced by the stomach, rises during caloric restriction [30]. Known as the “hunger hormone”, ghrelin acts on the hypothalamic arcuate nucleus, upregulating orexigenic neurons like neuropeptide Y (NPY) and agouti-related peptide (AgRP), which powerfully stimulate appetite. Ghrelin also sensitizes dopaminergic reward pathways, enhancing the appeal of high-calorie foods, especially under restrictive dietary conditions. Insulin levels decrease during caloric restriction, given the reduction in glucose availability and lower postprandial response. Reduced insulin leads to diminished activation of anorexigenic pathways in the hypothalamus, lowering satiety and enhancing the appetite-promoting effects of NPY and AgRP neurons [32]. Peptide YY (PYY) is secreted postprandially by the distal intestine in proportion to meal size and is known for promoting satiety. Caloric restriction lowers PYY release, weakening the post-meal satiety effect and enhancing the drive to eat. Glucagon-like peptide-1 (GLP-1), released from the intestinal L cells, normally enhances satiety, modulates insulin secretion, and slows gastric emptying. During caloric restriction, GLP-1 levels decrease, weakening satiety signals and reducing the insulinotropic effects that aid in glucose regulation. This adjustment heightens hunger and reduces the body’s ability to suppress further intake [32].

In a practical example, the study by Doucet et al. examined the effect of weight loss on appetite in 17 participants over a 15-week program followed by an 18-week maintenance phase. The results showed increased fasting hunger, desire to eat, and prospective food consumption after both phases, with correlations between cortisol changes and appetite measures in men (e.g., desire to eat, r = 0.67, *p* < 0.05) and women (e.g., desire to eat, r = 0.76, *p* < 0.05). Fasting cortisol emerged as the strongest predictor of appetite changes, with leptin also influencing fullness in men (r = 0.68, *p* < 0.05) during the maintenance phase [33]. Notably, as shown by the study of Sumithran et al., this increase in appetite persists in the long term after weight loss. This study investigated hormonal changes following diet-induced weight loss in 50 overweight or obese participants. Significant weight loss (mean 13.5 ± 0.5 kg) led to reductions in leptin, PYY, cholecystokinin (CCK), and insulin levels (*p* < 0.001), while ghrelin and gastric inhibitory polypeptide levels increased (*p* < 0.001). These hormonal changes, which promote appetite and weight regain, persisted one-year following weight loss, with levels of leptin (*p* < 0.001) and ghrelin (*p* < 0.001) remaining significantly altered, suggesting a physiological basis for the high rate of weight regain [34].

### 6.3. Lipolysis and Ketogenesis

With reduced caloric intake, the body shifts to fat mobilization as a primary energy source, promoting the breakdown of stored triglycerides and shifting to ketone production to preserve glucose. Hormone-sensitive lipase (HSL) activity increases in adipose tissue under caloric restriction as insulin levels decline. Glucagon and catecholamines stimulate HSL through β-adrenergic receptors, promoting lipolysis and releasing free fatty acids (FFAs) and glycerol into circulation. FFAs serve as primary fuel sources for muscle and liver, while glycerol is shuttled to the liver for gluconeogenesis [35].

As FFAs reach the liver, they undergo β-oxidation, generating acetyl-CoA. When acetyl-CoA production exceeds the Krebs cycle capacity because the intermediate oxalate is consumed in gluconeogenesis, it is diverted to ketogenesis, producing ketone bodies (acetoacetate, β-hydroxybutyrate, and acetone) as alternative fuels, especially critical for the brain. The shift to ketone utilization reduces glucose demand, preserving skeletal muscle by decreasing the need for protein breakdown. Ketogenesis is potentiated by low insulin and elevated glucagon, which together activate the transcription factors peroxisome proliferator-activated receptor alpha (PPAR-α) and forkhead box protein O1 (FOXO1), both of which drive ketogenic enzyme expression [36].

### 6.4. Muscle Protein Degradation

Caloric restriction triggers both muscle catabolism to supply amino acids for gluconeogenesis and hormone-mediated mechanisms to preserve lean mass. Cortisol rises in response to caloric restriction, promoting proteolysis in skeletal muscle. Cortisol activates proteolytic enzymes, including the ubiquitin–proteasome system and lysosomal pathways, to supply amino acids for gluconeogenesis, ensuring a steady glucose supply for glucose-dependent tissues [15]. Growth hormone (GH) levels may increase with caloric restriction, stimulating lipolysis and reducing muscle protein breakdown by inhibiting the effects of cortisol. GH activates lipolysis via β-adrenergic pathways, sparing muscle protein by directing energy production toward lipid oxidation [21]. Fluctuations in testosterone and estrogen can significantly influence muscle preservation. Testosterone stimulates myofibrillar protein synthesis (MPS) via mechanistic target of rapamycin (mTOR) activation, enhancing muscle anabolism and reducing protein breakdown, while estrogen modulates muscle sensitivity to insulin, supporting metabolic efficiency and indirectly preserving muscle tissue [23].

**Figure 1:**
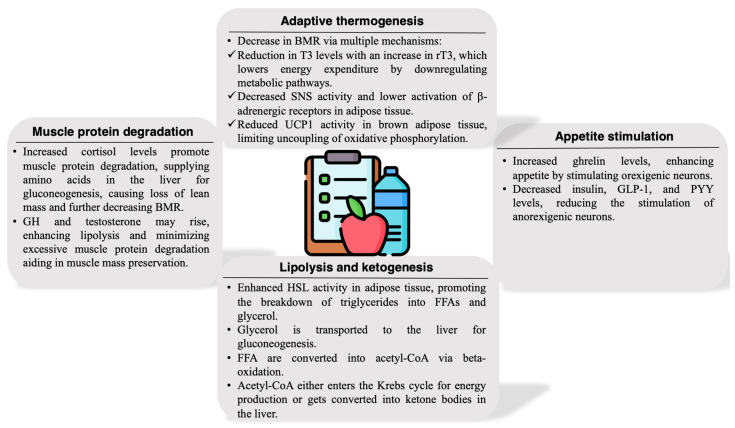
Key adaptations to caloric restriction affecting weight loss and body composition. Abbreviations. BMR (Basal Metabolic Rate); FFAs (Free Fatty Acids); GLP-1 (Glucagon-Like Peptide-1); GH (Growth Hormone); HSL (Hormone-Sensitive Lipase); PYY (Peptide YY); rT3 (Reverse Triiodothyronine); SNS (Sympathetic Nervous System); T3 (Triiodothyronine); UCP1 (Uncoupling Protein 1).

The key adaptations to caloric restriction affecting weight loss and body composition are illustrated in Figure 1.

## 7. Adaptations to Overfeeding

In conditions of caloric surplus, the body engages mechanisms that promote energy dissipation, modulate satiety, and facilitate energy storage. However, these responses vary significantly, influencing individual propensity toward weight gain or maintenance.

### 7.1. Luxurious Energy Expenditure

In contrast to adaptive thermogenesis following caloric restriction, overfeeding can lead to an increase in the BMR and RMR for some individuals, a phenomenon known as “luxurious energy expenditure”. This process allows the body to burn excess calories rather than store them, enabling some individuals to maintain a stable weight despite increased caloric intake. However, just as with caloric restriction, the degree of metabolic adaptation to overfeeding varies significantly among individuals, with some experiencing only minor increases in BMR, while others exhibit substantial metabolic acceleration [37].

Overfeeding stimulates a peripheral conversion of T4 to T3 via the enzyme type II iodothyronine deiodinase. Elevated T3 levels boost metabolic rate by increasing mitochondrial biogenesis and oxidative phosphorylation, enhancing thermogenesis. T3 also activates UCP1 in brown adipose tissue (BAT), directly stimulating thermogenesis [38].

The SNS increases in response to caloric surplus, with norepinephrine activating β-adrenergic receptors, particularly in BAT. This stimulates UCP1 in BAT mitochondria, generating heat through uncoupled respiration. Catecholamine release also supports lipolysis, preparing stored triglycerides for oxidation, though these fatty acids are rapidly metabolized in BAT [39].

Rising in proportion to body fat, leptin increases energy expenditure by stimulating sympathetic outflow and enhancing UCP1 expression in BAT. Leptin also augments fatty acid oxidation and reduces lipogenic (fat storage) pathways in peripheral tissues, limiting fat accumulation during overfeeding. However, in obesity with chronic hyperleptinemia, there is a development of leptin resistance, which prevents the stimulation of lipolysis and the suppression of appetite and leads to insulin resistance [19].

In a practical example of metabolic adaptation to overfeeding, Morrison et al. observed that with overfeeding (+1180 kcal/day), significant increases in body and fat mass (1.6 kg and 1.3 kg, respectively) were only noted after a 28-day period (*p* < 0.05). Additionally, postprandial glucose and insulin responses remained stable after 5 days but showed modest increases by 28 days, indicating some adaptation to energy surplus without immediate impairment in glucose regulation (*p* < 0.05) [40].

### 7.2. Appetite Suppression

Overfeeding triggers anorexigenic hormonal responses that limit further food intake by enhancing satiety. Elevated insulin levels postprandially increase glucose uptake and storage, reducing blood glucose and signaling satiety. Insulin acts centrally on the hypothalamus to inhibit NPY and AgRP, downregulating orexigenic signals and supporting reduced intake [32]. Overfeeding suppresses ghrelin production, limiting orexigenic signaling to the hypothalamus and decreasing hunger. Lower ghrelin levels reduce food-seeking behavior and reinforce satiety, helping to balance caloric intake. Released from intestinal L cells and I cells, GLP-1, PYY, and CCK rise with increased food intake, especially in response to fats and proteins. These hormones act on the hypothalamus to enhance satiety and delay gastric emptying, providing physiological signals that help regulate meal frequency and portion size in response to caloric excess [32].

### 7.3. Lipogenesis

Overfeeding promotes adipocyte expansion and differentiation, enhancing the body’s ability to store surplus energy and prevent ectopic fat accumulation in non-adipose tissues. Excess fats from the diet are stored primarily as triglycerides. Specifically, lipoprotein lipase (LPL) activity rises in adipose tissue in response to insulin and high energy intake. LPL hydrolyzes circulating triglycerides from chylomicrons and very-low-density lipoproteins into FFAs, which are then stored in adipocytes as triglycerides [41]. In addition to fats, surplus glucose or amino acids are directed to the adipose tissue, where they undergo de novo lipogenesis and are converted to fatty acids and subsequently to triglycerides [42]. This conversion reflects a key energy conservation mechanism, allowing the body to store excess carbohydrate-derived energy as fat.

In response to sustained overfeeding, preadipocytes differentiate into mature adipocytes, driven by transcription factors, such as proliferator-activated receptor gamma (PPARγ) and CCAAT/enhancer-binding protein alpha (C/EBPα), increasing fat storage capacity and mitigating metabolic risks associated with ectopic fat deposition in organs like the liver (non-alcoholic fatty liver disease) and muscle (myosteatosis) [43].

### 7.4. Muscle Protein Synthesis

Excess energy intake, particularly when combined with adequate protein intake, promotes muscle anabolism and protein synthesis, supported by anabolic hormones. Elevated insulin promotes amino acid uptake and protein synthesis in muscle, enhancing muscle mass, which in turn increases the BMR due to the higher metabolic demand of lean tissue [12]. GH and insulin-like growth factor I (IGF-I) are upregulated in response to caloric and protein surpluses, stimulating MPS and hypertrophy, particularly when combined with resistance exercise [21,22]. Furthermore, testosterone supports muscle growth by increasing MPS and reducing protein breakdown, contributing to increased lean mass and metabolic flexibility [23].

**Figure 2:**
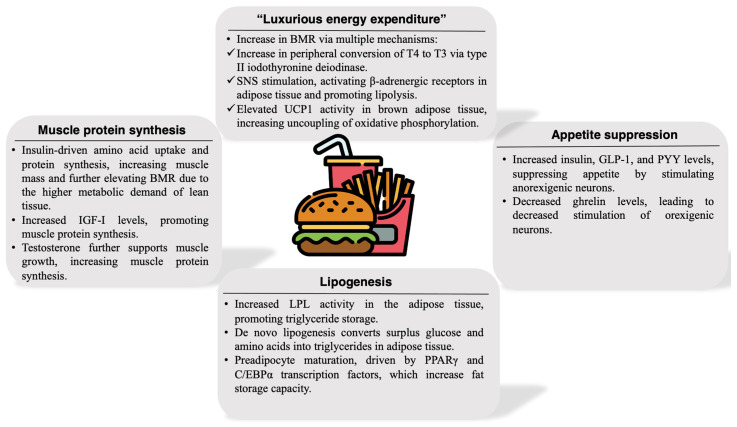
Key adaptations to overfeeding affecting weight gain and body composition. Abbreviations. BMR (Basal Metabolic Rate); C/EBPα (CCAAT/Enhancer-Binding Protein Alpha); GLP-1 (Glucagon-Like Peptide-1); IGF-I (Insulin-Like Growth Factor I); LPL (Lipoprotein Lipase); PPARγ (Peroxisome Proliferator-Activated Receptor Gamma); PYY (Peptide YY); SNS (Sympathetic Nervous System); T3 (Triiodothyronine); T4 (Thyroxine); UCP1 (Uncoupling Protein 1).

The key adaptations to overfeeding restriction affecting weight gain and body composition are illustrated in Figure 2.

## 8. Variability in Individual Responses to Diet

The variation in body composition changes—whether in terms of fat gain, muscle gain, fat loss, or muscle preservation—arises from a rich interplay of genetic predispositions, epigenetic modifications, behavioral influences, and environmental factors. Together, these layers contribute to a personalized metabolic response, providing insight into why individuals experience unique outcomes in response to similar caloric conditions.

### 8.1. Variability in Fat Gain During Overfeeding

#### 8.1.1. Genetic Polymorphisms Affecting Insulin Sensitivity and Lipid Metabolism

Genetic polymorphisms in key genes regulating insulin sensitivity and lipid metabolism significantly influence an individual’s efficiency in fat storage, particularly under conditions of caloric surplus. Variants in the *PPARG* gene can markedly alter the activity of PPARγ, a nuclear receptor that plays a crucial role in adipocyte differentiation and lipid storage. For instance, specific polymorphisms like *PPARG* Pro12Ala have been associated with variations in fat storage capacity and insulin sensitivity, making certain individuals more predisposed to adipose tissue expansion [44]. Similarly, polymorphisms in the *FABP4* gene, encoding fatty-acid-binding protein 4, modulate fatty acid transport and storage within adipocytes, further contributing to differences in lipid metabolism [45].

Beyond these genetic influences, the body’s response to insulin—a hormone central to glucose and lipid regulation—also exhibits notable variability among individuals. Insulin sensitivity is not solely determined by the density of insulin receptors (IRs) on adipocytes and muscle cells but is also shaped by the efficiency of post-receptor signaling pathways. When insulin binds to its receptor, it initiates a complex signaling cascade via IRS1 and IRS2 and the phosphoinositide 3-kinase/protein kinase B (PI3K-AKT) pathway, facilitating glucose uptake and lipid storage [46]. Variability in IRS phosphorylation, driven by specific polymorphisms, can alter the strength of these signals, ultimately impacting glucose disposal and lipid storage efficiency. For instance, individuals with polymorphisms in the *PI3K* gene may experience an enhanced or diminished insulin response, affecting their propensity for fat gain during periods of overfeeding [46]. Thus, genetic variations across these pathways underscore the personalized nature of metabolic responses to caloric excess and insulin action.

#### 8.1.2. Epigenetic Modifications in Lipogenesis and Lipolysis Pathways

Epigenetic modifications, including DNA methylation and histone acetylation, critically regulate gene expression in lipogenic and lipolytic pathways, influencing variability in fat storage and lipid metabolism. For instance, methylation of the lipoprotein lipase (*LPL*) gene promoter can alter *LPL* expression, impacting triglyceride uptake by adipocytes. High *LPL* activity enhances fat storage efficiency, while low activity limits fat uptake, contributing to differences in fat accumulation [47]. Additionally, histone acetylation near *SREBF1* (encoding sterol regulatory element-binding protein 1c, or SREBP-1c) affects lipogenic gene expression, with elevated acetylation promoting fatty acid synthesis, particularly during caloric surplus [48]. SREBP-1c, a key transcription factor, regulates genes involved in de novo lipogenesis and responds to high insulin and glucose levels during overfeeding by upregulating enzymes like acetyl-CoA carboxylase and fatty acid synthase (FAS), thereby converting dietary carbohydrates into stored fat. Variability in SREBP-1c activation can lead to differences in fatty acid synthesis rates and predisposition to fat storage. Additionally, genetic polymorphisms within *SREBF1* may alter SREBP-1c’s responsiveness to insulin and nutrient signals, further impacting lipogenesis efficiency [48]. Together, these epigenetic and genetic factors illustrate the complex regulation of lipid storage and synthesis, shaping individual differences in fat gain and metabolic responses to diet.

#### 8.1.3. Differences in Fat Storage Capacity and Adipocyte Differentiation

Adipocytes vary not only in size but also in their ability to proliferate and differentiate in response to caloric surplus. During overfeeding, PPARγ activation facilitates the differentiation of preadipocytes into mature adipocytes, increasing the body’s ability to store excess lipids. However, individual differences in PPARγ expression and responsiveness influence adipogenic potential, affecting whether surplus calories are efficiently stored in adipose tissue or lead to ectopic fat accumulation in non-adipose tissues like liver and muscle. Epigenetic modifications, such as methylation of the PPARγ promoter, can either enhance or inhibit this capacity, further influencing individual fat gain patterns [49].

#### 8.1.4. Variability in BAT Activity and UCP1 Expression

As already stated, BAT plays a crucial role in thermogenesis through the expression of UCP1, which dissipates energy as heat. Individuals with higher baseline BAT volume or greater UCP1 expression exhibit increased non-shivering thermogenesis during overfeeding, reducing the propensity for fat gain. UCP1 activation is driven by sympathetic stimulation through β-adrenergic receptors, and genetic variations in these receptors, especially adrenergic beta-3 receptors, influence BAT responsiveness to norepinephrine. People with more active BAT can expend excess calories more effectively, thus reducing fat storage [50]. Additionally, PR/SET domain 16 (PRDM16), a transcriptional regulator specific to BAT differentiation, may vary in expression among individuals, affecting their capacity for BAT-mediated thermogenesis [51].

#### 8.1.5. Impact of Lifestyle Factors

Lifestyle behaviors like exercise, stress management, and sleep quality significantly affect insulin sensitivity and lipid metabolism, modifying fat gain tendencies during overfeeding. Exercise increases insulin sensitivity through enhanced glucose transporter type 4 (GLUT4) translocation in muscle cells, redirecting glucose toward muscle rather than adipose tissue [52]. Additionally, chronic stress elevates cortisol, which can impair insulin sensitivity and promote visceral fat storage [53]. Poor sleep quality is linked to increased ghrelin levels and decreased leptin levels, enhancing hunger and potentially leading to higher caloric intake during overfeeding [54].

#### 8.1.6. Influence of Gut Microbiota

The gut microbiota, influenced by dietary patterns and lifestyle, significantly contribute to individual variability in fat gain. Certain microbial profiles efficiently harvest energy from food and produce short-chain fatty acids (SCFAs) like acetate, propionate, and butyrate, which can stimulate lipogenesis, particularly by activating SREBP-1c [49]. Diets rich in processed carbohydrates and saturated fats promote a microbiota composition that enhances energy harvesting, predisposing some individuals to greater fat accumulation. Conversely, fiber-rich diets support a more favorable microbiota that increases satiety and may limit fat gain during overfeeding. Emerging research highlights how microbiome composition affects SCFA production, influencing the extent to which overfeeding leads to fat storage. This dynamic between diet, microbiota, and host metabolism plays a crucial role in individual differences in fat gain [55].

### 8.2. Variability in Muscle Gain During Overfeeding

#### 8.2.1. Genetic Polymorphisms in Muscle Hypertrophy Pathways

Variants in genes governing MPS and hypertrophy influence muscle growth response to caloric surplus. The *IGF1* gene has polymorphisms such as *IGF1* CA-repeat variants, associated with differences in IGF-1 levels and muscle mass accretion [56]. Variability in GH receptor (GHR) sensitivity and IGF-1 production contributes to differences in muscle gain. Individuals with GHR polymorphisms that enhance receptor activity experience more pronounced muscle growth in response to GH, as IGF-1 stimulates MPS and reduces protein degradation [57]. Genetic factors affecting IGF-1 binding protein (IGFBP) levels, which regulate IGF-1 availability, further modulate muscle hypertrophy responses [58]. Furthermore, the AKT1 rs1130214 polymorphism affects the AKT pathway, a critical component of the mTOR signaling cascade, influencing MPS rates [59]. Satellite cells, which are muscle stem cells, are crucial for muscle hypertrophy, as they fuse to existing muscle fibers, increasing muscle mass. Variability in satellite cell activation and fusion capacity impacts muscle growth potential. Additionally, transcription factors like Pax7 that govern satellite cell activation exhibit differential expression, affecting individual muscle adaptation to caloric surplus [60]. Myostatin, a negative regulator of muscle growth, inhibits satellite cell proliferation; thus, individuals with lower myostatin levels or genetic variations (e.g., MSTN rs1805086 mutation) in myostatin activity experience greater muscle gains [61].

Muscle growth is heavily dependent on MPS, which is regulated by mTOR signaling and ribosomal biogenesis. Testosterone exerts anabolic effects through androgen receptor (AR) activation, promoting MPS. Differences in AR density and sensitivity modulate individual muscle gain capacity, with those exhibiting higher AR density responding more robustly to testosterone [23]. Furthermore, epigenetic modifications, such as acetylation of AR gene regions, can amplify AR expression, enhancing testosterone’s anabolic effects [62]. Variability in AR co-activators (e.g., SRC1, TIF2) also affects transcriptional regulation of muscle growth genes, contributing to differences in muscle hypertrophy [63]. Furthermore, during overfeeding, amino acid and insulin availability activates mTOR, stimulating MPS. Some individuals exhibit higher basal rates of MPS or enhanced sensitivity to mTOR stimulation due to genetic factors, such as polymorphisms in rs2295080 [64].

#### 8.2.2. Effects of Resistance Training on Muscle Growth

Resistance training enhances anabolic signaling and increases muscle hypertrophy when combined with overfeeding. The type, frequency, and intensity of exercise influence mTOR activation and satellite cell proliferation. High-volume resistance training induces a greater activation of the PI3K–AKT–mTOR pathway, increasing MPS rates and amplifying caloric surplus effects. According to a mechanistic study in mice, the increased sensitivity of mTORC1 to leucine persists for at least 48 h after exercise, indicating that the “anabolic window” for protein intake extends beyond the initial hours following exercise [65].

#### 8.2.3. Nutritional and Supplementation Influences on Muscle Hypertrophy

Adequate protein intake, particularly of leucine-rich sources, directly influences muscle hypertrophy by stimulating mTOR and MPS. A recent study explored how leucine and insulin activate mTOR signaling pathways in human skeletal muscle cells. Primary human myotubes were exposed to leucine, insulin, or both, and their effects on mTOR signaling, specifically on mTOR and p70S6K phosphorylation, were assessed over varying periods. The results showed that leucine independently and transiently stimulated mTOR signaling without affecting amino acid transporter gene expression. However, insulin also increased mTOR signaling and modified the gene expression of amino acid transporters. Notably, leucine enhanced the expression of the nutrient-sensing protein hVps34, which may regulate amino acid transport and mTOR activation in muscle tissue [66]. Despite these mechanistic insights, supplementation with leucin was not shown to significantly impact body composition and physical performance in humans [67].

### 8.3. Variability in Fat Loss During Caloric Restriction

#### 8.3.1. Adaptive Thermogenesis and Thyroid Hormone Responsiveness

As already analyzed, reductions in the BMR during caloric restriction, known as adaptive thermogenesis, vary widely due to differences in thyroid hormone metabolism. Lowered thyroid hormones, particularly T3, reduce metabolic rate by downregulating mitochondrial biogenesis and ATP turnover [25,26,30]. For instance, a single-nucleotide polymorphism in the type 2 deiodinase gene (p.Thr92Ala) has been found to be associated with hypertension, type 2 diabetes mellitus (T2DM), insulin resistance, and body mass index (BMI) [68]. Genetic variations in thyroid hormone receptors can alter sensitivity to T3, influencing how efficiently an individual can maintain energy expenditure and sustain fat loss during restriction [69].

#### 8.3.2. Genetic Polymorphisms and Epigenetic Modifications in Fat Oxidation and Lipolysis Pathways

The rate of lipolysis in adipose tissue during caloric restriction depends on the sensitivity of β-adrenergic receptors to catecholamines like norepinephrine. Genetic differences in β2-adrenergic receptor and β3-adrenergic receptor affect receptor responsiveness, modulating the rate at which stored triglycerides are hydrolyzed into FFAs. Individuals with heightened β-adrenergic sensitivity experience faster lipolysis, mobilizing fat stores more efficiently during restriction, whereas those with reduced receptor sensitivity may struggle to achieve the same level of fat oxidation. The β2-adrenergic receptor Arg16Gly and Gln27Glu polymorphisms alter β2-adrenergic receptor sensitivity, impacting lipolytic response to catecholamines. Individuals with high β-adrenergic sensitivity mobilize fat stores more effectively, supporting greater fat loss [70,71]. Polymorphisms in UCP2 and UCP3 affect mitochondrial efficiency, influencing the degree of energy dissipated as heat, and thus, the overall rate of fat oxidation [72].

Epigenetic modifications in genes governing lipolysis, such as HSL and adipose triglyceride lipase (ATGL), affect the ability to mobilize fat stores. For instance, methylation of the HSL promoter can downregulate HSL expression, reducing lipolytic activity and fat mobilization capacity [73]. Hypomethylation of the FOXO1 gene, a transcription factor promoting lipolysis, enhances fat oxidation, aiding in sustained fat loss. Epigenetic influences are also shaped by factors like exercise, which can reduce methylation of lipolytic genes and increase fat oxidation capacity [74].

#### 8.3.3. Ketogenic Adaptability and PPAR-α Activation

Ketone body production (ketogenesis) is crucial for energy provision during caloric restriction, as it reduces glucose demand and muscle breakdown. PPAR-α is a transcription factor that upregulates enzymes involved in ketogenesis, such as HMG-CoA synthase. Individuals with high PPAR-α expression or activity adapt more readily to fat oxidation, entering ketosis more efficiently. This adaptation spares glucose, facilitating sustained fat loss without the loss of lean mass. Polymorphisms in the *PPARA* gene can affect the degree of ketogenic adaptation, influencing fat oxidation rates during caloric deficit [75,76].

#### 8.3.4. The Role of Adiponectin in Fat Oxidation

Adiponectin, secreted by adipocytes, enhances fatty acid oxidation and improves insulin sensitivity. During caloric restriction, higher adiponectin levels promote fat mobilization and utilization in muscle tissue, contributing to sustained fat loss. A study has examined the relationship between BMI, adiponectin levels, and specific *ADIPOQ* gene polymorphisms (G276T and I164T) in young adult women in Jordan. Participants were categorized by BMI, and adiponectin levels were measured. The results showed that circulating adiponectin levels were inversely related to BMI, with lower levels in obese participants compared to other groups. However, there was no association between the *ADIPOQ* polymorphisms and BMI categories, suggesting that while adiponectin levels are linked to BMI, the *ADIPOQ* gene variants G276T and I164T may not significantly influence BMI or adiponectin levels in this population [77].

#### 8.3.5. Impact of Lifestyle Factors

Lifestyle factors like exercise intensity and type and macronutrient composition strongly influence fat loss. High-intensity interval training (HIIT) significantly promotes lipolysis, making it an effective strategy for reducing body fat and improving metabolic health. During HIIT, the intense bouts of activity trigger catecholamine release (epinephrine and norepinephrine), which binds to beta-adrenergic receptors on adipocytes, stimulating lipolytic enzymes like hormone-sensitive lipase (HSL) [78,79]. This initiates the breakdown of triglycerides into free fatty acids and glycerol, which are then released into the bloodstream for energy. The intermittent nature of HIIT—short bursts of intense work followed by recovery periods—creates a metabolic environment that maximizes fat oxidation, particularly after exercise. After HIIT, the body experiences an increase in excess post-exercise oxygen consumption (EPOC), which maintains elevated metabolic rates and fat oxidation levels for several hours, even in a resting state. HIIT has been shown to increase mitochondrial density and function within muscle cells, enhancing the muscles’ capacity to use free fatty acids for energy, especially in individuals with previously low aerobic capacity. This metabolic adaptation enables muscles to rely more on fat as an energy source during and after exercise, rather than glycogen. Additionally, HIIT has been associated with increased levels of adiponectin, an adipokine that regulates glucose levels and fatty acid breakdown, further supporting the role of HIIT in enhancing fat metabolism. These combined effects make HIIT a powerful tool for promoting lipolysis, reducing body fat, and improving overall metabolic health [78,79].

Furthermore, variations in dietary macronutrient composition impact fat loss and individual responses to diet due to complex, multifaceted interactions between metabolism, energy partitioning, and genetic factors. While the carbohydrate–insulin model posits that high-carbohydrate diets drive insulin secretion, leading to fat storage and weight gain, studies have challenged this by showing that weight loss success depends on broader factors beyond just carbohydrate intake [80]. Research has found that lower carbohydrate diets do not universally lead to greater long-term fat loss than higher carbohydrate diets. In controlled settings, high-carbohydrate and high-protein diets can improve satiety and metabolic balance, contributing to weight maintenance rather than fat gain. Furthermore, diets emphasizing whole grains, nuts, and vegetables—regardless of carbohydrate content—have shown associations with better weight maintenance compared to those high in refined sugars and saturated fats [80].

Chronic stress elevates cortisol, which promotes visceral fat storage and impairs lipolysis by increasing insulin resistance. Cortisol influences the hypothalamic–pituitary–adrenal axis, modulating the body’s metabolic response to caloric restriction. High cortisol sensitivity, due to polymorphisms in the glucocorticoid receptor gene, can predispose some individuals to greater fat retention during caloric restriction, particularly in the abdominal region [53].

### 8.4. Variability in Muscle Loss and Preservation During Caloric Restriction

#### 8.4.1. Differential Cortisol Responses and Proteolytic Activity

Cortisol, a catabolic hormone, plays a significant role in muscle proteolysis during caloric restriction. Individuals with heightened cortisol responses or polymorphisms in the glucocorticoid receptor (NR3C1) experience increased muscle breakdown, as cortisol promotes proteolytic enzyme activity [81]. Cortisol induces the expression of ubiquitin ligases like MuRF1 and Atrogin-1, which tag muscle proteins for degradation via the ubiquitin–proteasome pathway. Individuals with high sensitivity to glucocorticoids are more susceptible to muscle loss during caloric restriction, as elevated proteolytic activity accelerates muscle catabolism [82].

#### 8.4.2. Variability in AMPK Activation and Fat Oxidation

Adenosine monophosphate-activated protein kinase (AMPK) is activated under low energy states, enhancing fat oxidation and reducing reliance on muscle protein for energy. Individuals with high AMPK activation efficiently switch to fat as a fuel source, preserving muscle mass during restriction. Genetic variations in AMPK subunit genes (*PRKAA1*, *PRKAA2*) and epigenetic modifications affecting AMPK expression can influence the extent of this energy shift. Increased AMPK activity enhances mitochondrial biogenesis, supporting sustained fat oxidation and muscle preservation under caloric deficit [83,84].

#### 8.4.3. Muscle Fiber Type Composition and Myoglobin Levels

The proportion of oxidative (type I) versus glycolytic (type II) muscle fibers influences muscle preservation, as type I fibers are more resistant to proteolysis and rely heavily on fatty acid oxidation. Individuals with a higher proportion of type I fibers are less likely to experience muscle loss under caloric restriction, as these fibers utilize fat rather than amino acids for energy. Additionally, myoglobin-rich type I fibers facilitate oxygen delivery, optimizing oxidative metabolism and reducing protein breakdown. Genetic predispositions affecting fiber type composition and myoglobin expression contribute to variability in muscle loss susceptibility. In addition to genetic predisposition, exercise can significantly affect the distribution of fiber types [85].

#### 8.4.4. Influence of Sex Hormones on Muscle Preservation

Testosterone and estrogen play essential roles in muscle preservation, with testosterone promoting MPS via mTOR activation and estrogen supporting metabolic efficiency and glucose utilization. Men with higher baseline testosterone levels experience less muscle loss due to testosterone’s anti-catabolic effects. Furthermore, variations in the AR gene influence testosterone’s efficacy in maintaining lean mass, with certain polymorphisms enhancing muscle preservation during caloric restriction [23]. Women with higher estrogen levels benefit from estrogen’s mitochondrial protective effects, which enhance oxidative capacity and reduce proteolysis, as it reduces reliance on amino acids for gluconeogenesis. Polymorphisms in the estrogen receptor 1 (*ESR1*) gene influence estrogen sensitivity, affecting muscle retention capacity in women [86].

#### 8.4.5. Impact of Lifestyle Factors

Resistance exercise plays a fundamental role in preserving muscle mass during weight loss by activating the mTOR pathway and subsequently MPS. This pathway, particularly stimulated during resistance training, counteracts muscle proteolysis, or the breakdown of muscle proteins, which can occur when individuals are in a calorie deficit. Mechanistically, mTOR activation increases the translation of proteins necessary for muscle repair and growth, offsetting the catabolic processes typically triggered by caloric restriction [87].

High protein intake further supports muscle preservation by providing essential amino acids, particularly leucine, which is a powerful stimulator of the mTOR pathway. Leucine’s role is crucial, as it directly activates mTORC1 at the cellular level, enhancing the translation and assembly of muscle proteins, leading to more effective muscle maintenance or growth even during periods of reduced calorie intake [65]. Optimal protein intake for muscle preservation in energy restriction typically falls around 1.6–2.4 g/kg of body weight per day. In one study examining the effects of protein intake during a short-term caloric deficit, participants consuming a lower protein amount (1.0 g/kg of body weight daily) lost an average of 1.6 kg (3.5 pounds) of muscle, whereas those with a higher intake (2.3 g/kg daily) experienced a significantly smaller loss of only 0.3 kg (0.66 pounds) of muscle mass [88]. A similar study evaluated protein intakes of 0.8 g/kg, 1.6 g/kg, and 2.4 g/kg per day, revealing that both higher intake levels (1.6 and 2.4 g/kg) were more effective in preserving lean body mass compared to the lower 0.8 g/kg intake. Interestingly, no substantial additional benefit was observed at 2.4 g/kg over 1.6 g/kg [89].

Stress management is equally critical, as chronic stress elevates cortisol, a glucocorticoid hormone known to have catabolic effects on muscle tissue. Elevated cortisol levels enhance muscle protein breakdown by promoting proteolysis, particularly in skeletal muscle, to mobilize amino acids for gluconeogenesis in the liver. A Mendelian randomization study explored cortisol’s impact on muscle strength and mass, particularly in the context of sarcopenia, a muscle-wasting condition. Using genetic data from over 12,500 individuals, researchers identified three single-nucleotide polymorphisms associated with cortisol levels to investigate causal links with muscle metrics like grip strength and lean mass. The results indicate that higher cortisol levels are associated with reductions in both grip strength and lean body mass, especially in women, with fasting glucose emerging as a key mediating factor. The findings suggest that cortisol contributes to age-related sarcopenia by promoting protein degradation and inhibiting muscle synthesis, with elevated glucose levels possibly accelerating these effects [90].

Sleep significantly influences muscle preservation during caloric restriction. Adequate sleep supports recovery, optimizes hormone balance—particularly GH, which peaks during slow-wave sleep and is essential for muscle repair and regeneration—and enhances mTOR signaling and therefore MPS. Disrupted sleep, on the other hand, impairs GH secretion, limiting muscle repair and activating a catabolic state that makes muscle tissue more prone to breakdown [86]. This also blunts the mTOR pathway, as GH supports IGF-1 production, which is necessary for mTOR activation and MPS. Additionally, insufficient sleep elevates cortisol levels, further promoting muscle breakdown through increased proteolysis. By supporting GH and IGF-1 levels, quality sleep creates a hormonal environment favorable to muscle preservation, counteracting cortisol’s catabolic effects. Integrating sleep with resistance training, protein intake, and stress management offers a robust strategy to protect muscle mass during energy restriction [91].

In summary, the variability in individual responses to diet is driven by a complex interplay of genetic, epigenetic, hormonal, and lifestyle factors. Recognizing these differences emphasizes the need for personalized approaches in dietary interventions. Future research should focus on bridging these insights with clinical applications to develop precision strategies that account for individual metabolic profiles and long-term sustainability.

## 9. Precision Nutrition

Given the significant variability in individual responses to weight management interventions, a one-size-fits-all approach often falls short. Precision nutrition, as an integral part of precision medicine, addresses this variability by leveraging comprehensive metabolic, hormonal, and genetic data to create highly tailored dietary and lifestyle plans. This approach recognizes that individual factors—such as genetic polymorphisms, epigenetic modifications, and hormonal profiles—can profoundly influence metabolic responses to diet, caloric restriction, and exercise. Precision medicine uses these insights to identify personalized strategies, potentially overcoming adaptive mechanisms like metabolic slowdown or resistance to fat loss. By focusing on the individual’s unique metabolic profile, precision nutrition aligns dietary interventions with specific genetic and hormonal characteristics, allowing for sustainable and effective weight management. Recent precision-based trials underscore the efficacy of personalized weight loss interventions, providing promising data for future, individualized approaches. Here, we examine key examples and their applications in advancing weight management outcomes.

### 9.1. The DIETFITS Trial

The DIETFITS trial involving 609 overweight adults compared a healthy low-fat (HLF) diet with a healthy low-carbohydrate (HLC) diet for 12-month weight loss. The results showed no significant difference in weight change between the HLF (−5.3 kg) and HLC (−6.0 kg) groups (mean difference 0.7 kg; 95% CI, −0.2 to 1.6; *p* = 0.20). Additionally, no significant diet–genotype interaction (*p* = 0.20) or diet–insulin interaction (*p* = 0.47) was observed. Secondary outcomes favored HLF for low-density lipoprotein (−5.74 mg/dL; 95% CI, −9.38 to −2.09) and HLC for high-density lipoprotein cholesterol (2.24 mg/dL; 95% CI, −3.33 to −1.15) and triglycerides (−18.25 mg/dL; 95% CI, −28.84 to −7.65) [92].

### 9.2. The NOW Trial

The NOW randomized controlled trial (*n* = 140) compared a nutrigenomics-guided lifestyle intervention (GLB + NGx) to a population-based weight management program (GLB) for improving long-term dietary adherence. At 12 months, only the GLB + NGx group achieved a significant reduction in total fat intake from 36.0% ± 4.8% kcal to 30.2% ± 8.7% kcal (*p* = 0.02) and greater adherence to fat and saturated fat guidelines (*p* < 0.05). The study incorporated the theory of planned behavior to control confounders, suggesting that personalized, genetic-based advice enhances dietary change beyond traditional guidelines, supporting nutrigenomics as an effective tool for sustained weight management [93].

### 9.3. The PREDICT1 Study

The PREDICT1 study, involving 1002 UK participants and 100 US participants, investigated inter-individual variability in postprandial metabolic responses, highlighting how person-specific factors, such as the gut microbiome, influence these responses. The results showed substantial variation in postprandial triglyceride (103% coefficient of variation), glucose (68%), and insulin (59%) responses to identical meals. Person-specific factors like gut microbiome composition explained 7.1% of the variance in postprandial lipemia, more than meal macronutrients (3.6%). In contrast, meal macronutrients influenced postprandial glycemia (15.4%) more than gut microbiome factors (6.0%). Genetic factors showed a modest impact, explaining 9.5% of glycemic response variance (*p* < 0.05), with less influence on triglyceride (0.8%) and C-peptide responses (0.2%) [94].

The ML models developed from these data predicted triglyceride (r = 0.47, *p* < 0.001) and glycemic (r = 0.77, *p* < 0.001) responses, validated in the US cohort with similar accuracy (r = 0.42 for triglyceride, r = 0.75 for glucose). This study underscores significant variability in metabolic responses between individuals, even among twins, due to modifiable factors, which supports the potential for personalized nutrition strategies in reducing cardiometabolic risk [94].

### 9.4. BMR-Guided Nutrition Protocols

A systematic review and meta-analysis evaluated the impact of indirect calorimetry (IC)-guided energy delivery versus predictive equations in critically ill patients across eight RCTs (n = 911). IC-guided nutrition provided higher mean daily energy intake (622 kcal/day, *p* < 0.00001) and improved alignment with resting energy expenditure targets (89–106% in the IC group versus 56–79% in the control). IC-guided energy delivery significantly reduced short-term mortality (RR = 0.77; 95% CI 0.60 to 0.98; I2 = 3%, *p* = 0.03) compared to predictive equations. However, it did not significantly affect mechanical ventilation duration, ICU stay, hospital stay, or long-term mortality, suggesting IC’s utility in improving short-term outcomes [95].

## 10. AI and ML in Precision Weight Management

AI is revolutionizing weight management by addressing inter-individual variability through real-time, data-driven insights. Leveraging the power of M, these systems can integrate and analyze vast, complex datasets—including genomic, transcriptomic, metabolomic, hormonal, and environmental factors—to predict individual metabolic and behavioral responses to various dietary and lifestyle interventions. This capability enables the dynamic creation of tailored dietary and exercise plans that adapt continuously to the user’s physiological and metabolic states [96,97,98,99,100,101,102].

AI models have demonstrated remarkable potential in predicting metabolic responses, such as glycemic variability, to specific foods. For instance, the PREDICT1 study highlighted how AI can incorporate diverse factors, including gut microbiome composition, genetic variations, and dietary patterns, to develop personalized nutrition recommendations [94]. These models extend beyond traditional macronutrient calculations by considering hormone levels, metabolic flexibility, and energy partitioning, which are critical in addressing challenges like adaptive thermogenesis and hormonal dysregulation during weight loss.

AI-aided platforms optimize caloric intake and macronutrient distribution based on the user’s metabolic and hormonal profiles, addressing individual adaptive responses such as metabolic slowdown during caloric restriction. For example, ML algorithms can identify the specific macronutrient ratios that minimize hunger while maximizing fat oxidation and muscle preservation, thereby enhancing the efficacy of precision nutrition strategies. These algorithms can also incorporate circadian rhythms, physical activity levels, and psychological stressors to refine the recommendations further [96,97,98,99,100,101,102].

One of AI’s most transformative features is its ability to provide continuous, real-time feedback through wearable devices, mobile applications, and connected health platforms. By analyzing biometrics such as heart rate variability, blood glucose levels, and caloric expenditure, AI tools empower users to make immediate adjustments to their behavior, dietary intake, or physical activity. For instance, an AI-powered app may recommend a protein-rich snack after detecting a post-exercise drop in blood glucose, ensuring sustained energy levels and muscle recovery [96,97,98,99,100,101,102].

AI platforms also integrate behavioral psychology principles, tracking adherence trends and identifying factors contributing to deviations. Through predictive analytics, these systems can anticipate challenges such as periods of low motivation or high stress, offering proactive interventions to maintain consistency. This real-time adaptability enhances long-term success and adherence to personalized weight management plans [96,97,98,99,100,101,102].

The synergy between AI and emerging technologies is paving the way for unprecedented personalization in weight management. Wearable devices, such as continuous glucose monitors (CGMs) and smartwatches, provide real-time physiological data, while advanced biomarkers, including metabolomic and proteomic profiles, offer deeper insights into metabolic states. AI can integrate these data streams to refine predictive models, enabling a dynamic, iterative approach to weight management that evolves alongside the user’s changing physiology. For example, AI can adjust meal timing and composition based on detected variations in metabolic rate or hormonal fluctuations [96,97,98,99,100,101,102].

Despite its transformative potential, the efficacy of AI-based weight management systems must be rigorously validated through large-scale, randomized clinical trials. Future research should focus on assessing these systems’ ability to sustain long-term weight loss, improve adherence, and mitigate adaptive metabolic responses. Additionally, the ethical implications for data privacy and accessibility must be addressed to ensure equitable adoption across diverse populations. Advances in explainable AI (XAI) will also play a critical role in enhancing transparency, fostering trust, and promoting widespread clinical adoption of AI-driven solutions [95,96,97,98,99,100,101].

## 11. Limitations

This review synthesized diverse findings on metabolic and hormonal variability in weight management; however, certain limitations should be noted:

▪ Heterogeneity in Study Designs: A significant portion of the included studies rely on observational designs or small-scale trials, which limit causal inference and generalizability. The variability in methodologies—such as inconsistencies in defining and measuring BMR, hormonal fluctuations, and energy expenditure—makes direct comparison across studies challenging.
▪ Population Bias: Many studies are skewed toward specific populations, such as individuals with obesity or metabolic syndrome, with limited representation of other subgroups (e.g., athletes, older adults with sarcopenia, or individuals from diverse ethnic backgrounds). This limits the applicability of findings to broader populations.
▪ Inadequate Control for Confounders: Many studies fail to adequately control for confounding factors, such as sleep quality, stress levels, or dietary composition, which significantly influence metabolic responses and hormonal adaptations.
▪ Underexplored Environmental and Epigenetic Influences: While the role of genetic predispositions is often addressed, environmental and epigenetic influences, such as dietary patterns, socioeconomic status, and early-life exposures, are underexplored in the context of long-term weight management outcomes.
▪ Precision Medicine Challenges: Despite promising advances in precision approaches, such as nutrigenomics and ML models, most studies are pilot-scale and lack validation in larger, diverse cohorts. Furthermore, these approaches often suffer from high costs, which can hinder practical implementation in clinical settings.
▪ Focus on Short-Term Outcomes: The majority of the literature emphasizes short-term interventions, such as caloric restriction or overfeeding studies, without sufficient exploration of long-term sustainability and the persistence of metabolic adaptations over time.

Future research should prioritize large-scale, longitudinal studies with standardized methodologies and diverse populations to validate findings and refine precision strategies for sustainable weight management.

## 12. Future Directions and Practical Applications

### 12.1. Practical Applications

While hypocaloric diets are a cornerstone of obesity management, they often fail to address the adaptive responses that hinder long-term success, such as metabolic slowdown and hormonal dysregulation. Precision nutrition, which accounts for individual metabolic profiles, offers a more sustainable alternative. This approach emphasizes the quality of calories, the timing of meals, and the balance of macronutrients to enhance metabolic flexibility and prevent weight regain.

RMR-driven nutrition is an advanced approach that leverages indirect calorimetry to calculate an individual’s RMR accurately. By continuously monitoring how RMR changes with nutritional and exercise interventions, caloric intake can be precisely adjusted to meet metabolic demands. Additionally, calculating the respiratory exchange ratio (RER) alongside urine nitrogen excretion enables a detailed analysis of macronutrient utilization—determining the proportion of energy derived from fat, protein (indicating potential muscle loss), and carbohydrates. These data refine dietary and exercise strategies, targeting specific outcomes, such as fat loss or muscle gain. These techniques create a robust platform for precision nutrition and enable tailored follow-ups, ensuring that interventions are dynamically aligned with individual goals [95].

Ergospirometry is another valuable tool for maximizing fat loss through tailored exercise prescriptions. By identifying the optimal exercise intensity, where the RER is closest to 0.8, this approach ensures the highest reliance on fat oxidation during aerobic activity. Integrating these data with precision nutrition plans enhances the effectiveness of weight management strategies by promoting maximal fat loss while preserving lean muscle mass [103].

Strength training should be recognized as a foundational component of obesity prevention and management due to its multifaceted benefits for metabolic health. By increasing lean muscle mass, strength training elevates the RMR, leading to greater caloric expenditure even at rest. This increase in muscle mass also counteracts metabolic adaptations, such as the reduction in energy expenditure often observed during caloric restriction, thereby enhancing the sustainability of weight loss efforts. At the molecular level, resistance exercise improves glycolipid metabolism and mitochondrial biogenesis through pathways such as the miR-30d-5p/SIRT1/PGC-1α axis, as evidenced by studies in type 2 diabetes mellitus (T2DM) models. Lifang Zheng et al. demonstrated that resistance exercise significantly improves glucose homeostasis, insulin signaling, and mitochondrial function in T2DM mice by downregulating miR-30d-5p, which targets SIRT1 and impacts PGC-1α expression. These findings underscore the role of strength training in not only reducing lipid accumulation and enhancing fatty acid oxidation but also in regulating key molecular pathways that mitigate insulin resistance [104].

For older populations, strength training is especially valuable in preventing sarcopenia, the age-related loss of muscle mass and function. By preserving and increasing muscle mass, strength training not only supports metabolic health but also improves physical performance and reduces the risk of falls and frailty. When combined with aerobic exercise, this dual-modality approach maximizes fat oxidation, enhances cardiovascular health, and creates a synergistic effect that optimizes overall metabolic flexibility [105].

Emerging evidence suggests that targeting pre-obesity—defined as an intermediate stage with a BMI nearing 25–29.9 kg/m^2^—offers a critical window for intervention. Lifestyle interventions focusing on small, sustainable changes in dietary habits, physical activity, and stress management should be emphasized. For instance, incorporating resistance training, low-glycemic-index diets, and adequate sleep can prevent metabolic derangements associated with obesity. Beyond dietary and exercise interventions, holistic approaches that include mental health support, stress reduction, and sleep hygiene are essential. Stress management techniques, such as mindfulness and cognitive behavioral therapy, can reduce cortisol-induced weight gain, while improving sleep quality optimizes hormonal regulation and metabolism [106].

At the community level, public health strategies should prioritize creating environments that support healthy behaviors. Access to affordable, nutrient-dense foods, safe spaces for physical activity, and stress reduction programs must be integrated into broader public health initiatives to address the systemic challenges of obesity prevention.

AI-powered platforms, wearable devices, and continuous glucose monitors should be integrated into routine care to provide real-time feedback and personalized recommendations. These tools enhance adherence by tracking biometrics and offering actionable insights to users, making weight management dynamic and adaptive to individual needs [96,97,98,99,100,101,102].

By shifting the focus from reactive obesity treatment to proactive prevention and personalized care, these strategies provide a roadmap for tackling obesity and its associated challenges in a sustainable and effective manner.

### 12.2. Future Research Directions

Future research should prioritize large-scale, longitudinal studies to capture the long-term trajectory of metabolic and hormonal adaptations to various weight management interventions, with a particular emphasis on intervention sustainability, the persistence of metabolic adaptations, and their implications for weight regain or maintenance. By enrolling diverse populations with varying genetic, epigenetic, and cultural backgrounds, these investigations can establish a more nuanced understanding of the factors that influence long-term outcomes, thereby informing more inclusive and universally applicable strategies.

Advancements in multi-omics research—integrating genomics, metabolomics, proteomics, and microbiomics—hold promise for unraveling the complexity of individual responses to diet and exercise. Future work should delve deeper into hormonal regulation, such as the dynamics of leptin and ghrelin sensitivity, and epigenetic modifications, including DNA methylation in lipogenic pathways [97,107]. These efforts will clarify how genetic and epigenetic landscapes intersect with hormonal signaling to shape metabolic adaptability, ultimately guiding the development of more targeted interventions.

The incorporation of artificial intelligence and ML into precision nutrition research should be expanded and refined, with an eye toward validating predictive models in real-world settings. By leveraging longitudinal data from wearable sensors, mobile health applications, and clinical assessments, researchers can tailor weight management approaches in real time. More transparent and explainable AI models will be essential to foster trust among healthcare providers and patients, ensuring that these tools can be seamlessly integrated into clinical practice.

Future studies must also consider the interplay of lifestyle factors—such as exercise modalities, sleep patterns, and stress management techniques—and their influence on metabolic and hormonal environments. Understanding these modifiable components can enhance intervention efficacy and adherence, translating mechanistic insights into lasting, population-level improvements. As such, embracing a holistic research agenda that spans biological, behavioral, and environmental domains is key to realizing the full potential of precision nutrition in weight management.

## 13. Conclusions

In conclusion, this state-of-the-art review examined the metabolic and hormonal mechanisms that drive variability in weight management outcomes, highlighting the complexity of individual responses to caloric restriction and overfeeding. Key adaptive processes, including changes in the RMR, thyroid hormone regulation, SNS activity, and appetite modulation, were discussed at both the cellular and systemic levels. These findings underscore the intricate interplay of molecular and hormonal pathways in shaping weight outcomes, challenging the conventional “calories in, calories out” model.

By integrating these insights with precision nutrition approaches and emerging technologies, such as nutrigenomics, ML, and indirect calorimetry, this review proposes a novel framework for tailoring interventions to each individual’s unique metabolic profile. This approach not only addresses the limitations of traditional weight management paradigms but also provides a pathway for sustainable and effective strategies that are grounded in molecular biology and hormonal physiology. Future research should focus on bridging molecular insights with clinical practice to refine personalized interventions and optimize long-term weight management outcomes.

## Figures and Tables

**Table 1:** Head-to-head comparison of the key features of the “calories in, calories out” and carbohydrate–insulin models.

Feature	“Calories In, Calories Out”	Carbohydrate–Insulin Model	References
Core Concept	Weight change is governed by energy balance, where weight loss occurs by burning more calories than consumed.	Emphasizes calorie quality over quantity, suggesting that carbohydrate intake influences insulin response and fat storage.	[2,5,6,7]
Primary Focus	Energy balance (calories consumed versus calories burned).	Impact of carbohydrate types on insulin and subsequent fat storage.	[2,5,6,7]
Mechanism of Weight Gain	Excess caloric intake relative to expenditure results in weight gain.	High carbohydrate intake, especially high-glycemic-index carbs, elevates insulin, promoting fat storage.	[2,5,6,7]
Role of Macronutrients	All macronutrients contribute equally to total caloric intake; their effect on metabolism is not emphasized.	Carbohydrates, particularly refined ones, impact insulin levels, thereby affecting metabolic and hormonal responses.	[2,5,6,7]
Hunger and Satiety Regulation	Hunger is a byproduct of caloric deficit; it does not fully account for hormonal influences on appetite.	Insulin response from high carbohydrate intake can create cycles of hunger and satiety, potentially leading to overeating.	[2,5,6,7]
Impact on Fat Storage	Fat storage is viewed as a result of caloric surplus.	Insulin promotes lipogenesis (fat storage), particularly after high-carbohydrate meals.	[2,5,6,7]
Effectiveness of Low-Carb Diets	Not specifically emphasized; all caloric deficits can theoretically induce weight loss.	Low-carb diets reduce insulin levels, potentially reducing fat storage and increasing fat mobilization.	[2,5,6,7]
Metabolic Adaptations	Assumes a direct, linear relationship between caloric intake, expenditure, and weight change.	Recognizes adaptive mechanisms where high insulin levels can limit fat mobilization, influencing weight plateau or gain.	[2,5,6,7]
Application in Diet Planning	Caloric tracking and balance are prioritized; diet quality is less emphasized.	Focuses on carbohydrate quality and glycemic index to modulate insulin and manage body fat.	[2,5,6,7]
Limitations	Simplistic; may overlook inaccuracies in food labels, metabolic adaptations, and hormonal influences.	May not fully explain weight loss variability across individuals; less focus on caloric balance in overall diet.	[2,3,4,5,6,7]

**Table 2:** Key effects of major hormones affecting the metabolism of carbohydrates, fats, and proteins.

Hormone	Carbohydrate Metabolism	Fat Metabolism	Protein Metabolism	References
Insulin	Promotes glycolysis, facilitating energy utilization Increases glucose uptake in muscle and adipose tissue via GLUT4 translocation Inhibits hepatic gluconeogenesis Promotes glycogen synthesis and inhibits glycogenolysis in liver and muscle	Inhibits lipolysis and stimulates lipogenesis and triglyceride storage in the adipose tissue Stimulates fatty acid synthesis Inhibits hepatic ketogenesis	Increases amino acid uptake in muscle cells Inhibits proteolysis Stimulates MPS via mTOR signaling and other mechanisms	[12]
Glucagon	Inhibits hepatic glycolysis, ensuring glucose availability in the blood Stimulates hepatic gluconeogenesis Promotes glycogenolysis and inhibits glycogen synthesis in liver and muscle	Inhibits lipogenesis and stimulates lipolysis and free fatty acid liberation from the adipose tissue Stimulates beta-oxidation of fatty acids Promotes hepatic ketogenesis	Limited role in protein metabolism	[13]
Catecholamines	Promote glycogenolysis and inhibit glycogen synthesis in liver and muscle Stimulate hepatic gluconeogenesis Inhibit insulin release	Inhibit lipogenesis and stimulate lipolysis and free fatty acid liberation from the adipose tissue Stimulate beta-oxidation of fatty acids	Minimal impact on protein metabolism; slight increase in muscle proteolysis	[14]
Cortisol	Stimulates hepatic gluconeogenesis Reduces peripheral glucose uptake Decreases insulin sensitivity	Inhibits lipogenesis and stimulates lipolysis and free fatty acid liberation from the adipose tissue Can lead to central obesity if chronically elevated due to increased visceral fat deposition	Increases proteolysis, mobilizing amino acids for gluconeogenesis Reduces MPS during prolonged stress Leads to muscle wasting if chronically elevated	[15]
Thyroid Hormones	Stimulate glycolysis, glycogenolysis, gluconeogenesis, and oxidative phosphorylation	Stimulate both lipogenesis and lipolysis Stimulate beta-oxidation of fatty acids Increase thermogenesis	Enhance protein turnover by increasing both synthesis and degradation	[16]
Incretins	Enhance glucose-stimulated insulin secretion from pancreatic β cells Modulate postprandial glucose levels by slowing gastric emptying Inhibit glucagon release	Limited direct effect on fat metabolism	Limited direct effect on protein metabolism	[17]
Ghrelin	Increases hepatic gluconeogenesis indirectly by stimulating GH secretion Variable effects on insulin sensitivity	Limited direct effect on fat metabolism	Increases MPS primarily by stimulating GH secretion	[18]
Leptin	Inhibits hepatic gluconeogenesis Increases insulin sensitivity	Milnor stimulator of lipolysis and liberation of fatty acids from adipose tissue	Inhibits muscle proteolysis	[19]
Adiponectin	Inhibits hepatic gluconeogenesis Increases insulin sensitivity	Increases fatty acid beta-oxidation	Inhibits muscle proteolysis	[20]
GH	Stimulates hepatic gluconeogenesis Reduces peripheral glucose uptake Decreases insulin sensitivity	Inhibits lipogenesis and stimulates lipolysis and liberation of fatty acids from adipose tissue Increases fatty acid beta-oxidation	Promotes MPS and inhibits muscle proteolysis	[21]
IGF-I	Inhibits hepatic gluconeogenesis Increases peripheral glucose uptake Improves insulin sensitivity	Limited direct effect on fat metabolism	Promotes MPS and inhibits muscle proteolysis	[22]
Sex Steroid Hormones	Limited direct effect on carbohydrate metabolism Testosterone has been shown to influence insulin sensitivity in muscle	Testosterone inhibits lipogenesis and stimulates lipolysis and liberation of fatty acids from adipose tissue Estrogens promote subcutaneous fat distribution	Testosterone strongly stimulates MPS Estrogens have a mild stimulatory effect on MPS	[23,24]

Abbreviations. GH (Growth Hormone); GLUT4 (Glucose Transporter Type 4); IGF-I (Insulin-Like Growth Factor I); mTOR (Mechanistic Target of Rapamycin); MPS (Myofibrillar Protein Synthesis).
